# Editorial: The relationship between puberty and immune-driven disease

**DOI:** 10.3389/fped.2023.1244240

**Published:** 2023-07-06

**Authors:** Jonatan Leffler, Paola Triggianese

**Affiliations:** ^1^ Telethon Kids Institute, University of Western Australia, Perth, WA, Australia; ^2^ Rheumatology Allergology and Clinical Immunology, Department of Systems Medicine, University of Rome Tor Vergata, Rome, Italy

**Keywords:** autoimmunity, immune modulation, inflammation, puberty, sex hormones

Editorial on the Research Topic The relationship between puberty and immune-driven disease

The way the immune system operates differs between males and females. This is due to both differential expression of immune-related genes from the sex chromosomes as well as the immune modulatory properties of sex hormones. Together, these effects contribute to a skewed prevalence of disease and disease course between males and females, including allergic-, infectious-, autoimmune-, and cancerous disease (1, 2). The impact of sex hormones is evident across several disorders, including immune-driven pathologies such as asthma and multiple sclerosis (MS), which changes significantly in both prevalence and phenotype at the time of puberty (3, 4). As a result, puberty is a period where susceptibility to disease may dramatically change for an individual. It may also impact individuals with an already established disease who may face a transition in disease phenotype or severity, as demonstrated by this research topic. An established disease may also resolve at the time of puberty; a common example of this is allergic asthma, which decreases significantly in prevalence in males whilst the prevalence in females increases (3).

For this Research Topic we encouraged submission of studies on the topic of how immune-driven disease is influenced by puberty. We have included both original articles and reviews on disease that, after puberty, might be different in females and males, likely due to the effects of sex hormones (5).

Two reviews were included in this topic. One focused on the impact of puberty on autoimmune diseases in general and the impact of hormones on immune cells subsets, and another review focused specifically on the impact of puberty on MS. The first review by Yang et al. linked puberty to altered metabolism and levels of sex hormones, and evaluated how this modulated the immune system. This was related to pathogenesis of autoimmune conditions, such as systemic lupus erythematosus (SLE) and rheumatoid arthritis, of which some can have an early onset, even prior to puberty.

The second review by Ucciferri et al. focused specifically on the impact of puberty on MS. It highlighted the likely contribution of sex hormones to the development of pediatric MS through the observation that pre-puberty, the number of females with MS was the same as the number of males, whereas after puberty, MS was two to three times more common in females compared to males. The review also discussed the impact of BMI, puberty timing, and assisted reproductive techniques on the risk of developing MS. In addition, findings from animal models of MS, where the impact of puberty can be investigated in more detail, were also discussed and complemented with references to detailed immunological studies carried out in humans before and after puberty.

In addition to the reviews, our Research Topic also included original research focusing on the impact of puberty on defined autoimmune diseases such as thyroiditis as well as in the context of rare diseases related to abnormal complement system regulation, specifically hereditary angioedema due to C1-inhibitor deficiency (C1INH-HAE). The study by Kyristi et al. reported that in girls with premature puberty, a quarter of individuals also presented with autoimmune thyroiditis. From the study it was not clear if the premature puberty contributed to the autoimmune condition or if the thyroiditis instead contributed to premature puberty. A clear association with obesity and insulin resistance was interestingly observed. These findings illustrate that puberty may not only influence immune-driven disease, but puberty may be influenced by disease as well. The second study by Cancian et al. investigated the role of puberty on disease course and severity in Italian C1INH-HAE patients. Using questionnaires across centers in Italy, the study reported a significant increase in angioedema attacks following puberty, and that this worsening appeared more pronounced in females compared to males. The possible reason for this may be related to the impact of sex hormones since estrogens are well known to trigger angioedema attacks, which may also be important to consider during pregnancy (6).

Together these studies demonstrate the significant impact that puberty and sex hormones may have on immune-driven diseases. As these effects can both be positive and negative, this may provide an opportunity for novel and exciting therapeutic interventions aimed at modulating the immunological effects of sex hormones. Pilot studies of hormone supplementation in some autoimmune diseases have showed some promise as reviewed (7), but interventions using hormones may not be suitable for individuals of fertile age. Directly targeting the immune system may be a more appropriate strategy for such population. The immune modulatory effects of hormones are also relevant for transgender individuals who choose to take gender affirming hormone therapy to align their body with their gender. How exogenous sex hormones interact with male/female genes is an area where more immunology-focused research is required, as we have recently discussed (8).

In summary, puberty is a period of change in the mental and physiological aspects of an individual. As this research topic has also highlighted, significant changes also occur in the immune system, and these changes are important to consider when evaluating treatment strategies and clinical care for young individuals with immune-driven disease.
